# Sequence Determinants Spanning −10 Motif and Spacer Region Implicated in Unique *Ehrlichia chaffeensis* Sigma 32-Dependent Promoter Activity of *dnaK* Gene

**DOI:** 10.3389/fmicb.2019.01772

**Published:** 2019-08-02

**Authors:** Huitao Liu, Roman R. Ganta

**Affiliations:** Center of Excellence for Vector-Borne Diseases, Department of Diagnostic Medicine/Pathobiology, College of Veterinary Medicine, Kansas State University, Manhattan, KS, United States

**Keywords:** gene regulation, intracellular bacteria, *Anaplasmataceae*, sigma factor, *Ehrlichia chaffeensis*, RNA polymerase

## Abstract

*Ehrlichia chaffeensis* is an obligate intracellular tick-borne bacterium that causes human monocytic ehrlichiosis. Studying *Ehrlichia* gene regulation is challenge, as this and related rickettsiales lack natural plasmids and mutagenesis experiments are of a limited scope. *E. chaffeensis* contains only two sigma factors, σ^32^ and σ^70^. We previously developed *Escherichia coli* surrogate system to study transcriptional regulation from RNA polymerase (RNAP) containing *Ehrlichia* σ^32^ or σ^70^. We reported that RNAP binding motifs of *E. chaffeensis* genes recognized by σ^32^ or σ^70^ share extensive homology and that transcription may be initiated by either one of the sigma factors, although transcriptional efficiencies differ. In the current study, we investigated mapping the *E. chaffeensis dnaK* gene promoter using the pathogen σ^32^ expressed in *E. coli* lacking its native σ^32^. The *E. coli* surrogate system and our previously described *in vitro* transcription system aided in defining the unique −10 motif and spacer sequence of the *dnaK* promoter. We also mapped σ^32^ amino acids/domains engaged in its promoter regulation in *E. chaffeensis*. The data reported in this study demonstrate that the −10 and −35 motifs and spacer sequence located between the two motifs of *dnaK* promoter are critical for the RNAP function. Further, we mapped the importance of all six nucleotide positions of the −10 motif and identified critical determinants within it. In addition, we reported that the lack of C-rich sequence upstream to the −10 motif is unique in driving the pathogen-specific transcription by its σ^32^ from *dnaK* gene promoter. This is the first study in defining an *E. chaffeensis* σ^32^-dependent promoter and it offers insights about how this and other related rickettsial pathogens regulate stress response genes.

## Introduction

*Ehrlichia chaffeensis*, a Gram-negative, obligate intracellular tick-borne rickettsial pathogen, causes human monocytic ehrlichiosis (HME) (Dunning Hotopp et al., 2006). HME as an emerging infectious disease, first reported in the United States in 1987, has become one of the most prevalent tick borne diseases in the United States and is also described from several other parts of the world (Ismail et al., 2010; Yabsley, 2010). Further, *E. chaffeensis* infects several other vertebrates, including dogs, goats, coyotes and white-tailed deer (Dawson et al., 1996; Lockhart et al., 1997; Breitschwerdt et al., 1998; Dugan et al., 2000; Kocan et al., 2000; Davidson et al., 2001). The pathogen infection in people may result in an acute flu-like illness with symptoms ranging from persistent fever, headache, myalgia, anorexia and chills (Walker et al., 2008). HME patients may exhibit leukopenia, thrombocytopenia, anemia, and upgraded levels of serum hepatic aminotransferases (Walker et al., 2008). In addition, other related *Anaplasmataceae* family pathogens included in the genera *Ehrlichia* and *Anaplasma* have been established as causative agents of emerging diseases in people and various vertebrate animals in recent years (Walker and Dumler, 1996; Walker et al., 2008; Rikihisa, 2010). Though some progress is made in establishing genetics in *E. chaffeensis* (Cheng et al., 2013; Wang et al., 2017). and similarly in other related *Ehrlichia* and *Anaplasma* (Long et al., 2005; Felsheim et al., 2006; Crosby et al., 2014; Wood et al., 2014; mcClure et al., 2017), the genetic tool kit and its application is still limited. For example, it is not possible to investigate regulation of gene expression by transforming this group of important pathogens, possibly also because the pathogens lack naturally existing extrachromosomal plasmids. This major impediment limits the understanding of molecular mechanisms used by the pathogens in regulating gene expression in support of their continued survival in vertebrate and tick hosts and in causing pathogenesis (Dumler et al., 1993; Davidson et al., 2001; Unver et al., 2002). Several prior studies reported differences in gene expression of *E. chaffeensis* impacted by different host environments (Seo et al., 2008; Kuriakose et al., 2011). However, it is unclear how the organism regulates its gene expression in support of its adaptation to the hosts.

Regulation of gene expression in bacteria is primarily controlled at the transcription. An RNA polymerase (RNAP) core enzyme with a sigma (σ) factor offers a simple and valid mechanism for bacteria to rapidly accommodate to diverse environmental changes by suitably modifying the transcriptional profiles (Gruber and Gross, 2003; Gunesekere et al., 2006; Browning and Busby, 2016). Typically, an RNAP holoenzyme is a multi-subunit complex consists of a core enzyme containing two alpha (α), a beta (β), a beta′ (β′), and a omega (ω) subunits and then the inclusion of a σ factor (Chamberlin et al., 1983). A σ factor enables a core enzyme in specific binding to the promoter region of a gene for initiating transcription. Numbers of σ factors differ depending on the genome size variations and the environmental diversification of a bacterium (Kill et al., 2005). For example, *Escherichia coli* possesses seven σ factors, while 109 σ factors are identified in *Sorangium cellulosum* (Han et al., 2013; Tripathi et al., 2014). Obligate intracellular bacteria generally tend to have reduced genomes and consequently also have fewer σ factors (Darby et al., 2007). For example, *E. chaffeensis* genome of 1,176 kb has only two σ factor genes; *rpoD* (ECH_0760) (the primary housekeeping σ^70^ gene) and *rpoH* (ECH_0655) (the alternate σ^32^ gene) (Dunning Hotopp et al., 2006) (GenBank # NC_007799.1).

To study gene regulation in *E. chaffeensis*, we previously described *in vitro* transcription system and an *E. coli* surrogate system that is valuable in investigating gene regulation driven from its σ^70^ (Faburay et al., 2011; Liu et al., 2013, 2016). Earlier, we also defined the promoters of several pathogen genes by utilizing *in vitro* transcription assays where *E. coli* RNAP core enzyme is reconstituted with the recombinant *E. chaffeensis* σ factors (Faburay et al., 2011; Liu et al., 2013). Our studies demonstrated that the RNAP binding motifs (−10 and −35 regions) of *E. chaffeensis* gene promoters share extensive homology and that they are recognizable by RNAP with either one of its only two sigma factors; σ^32^ or σ^70^, although affinities vary for different gene promoters (Liu et al., 2013). In *E. coli*, gene expression of heat shock proteins; Dnak-DnaJ-GrpE and GroES-GroEL chaperone complexes, are controlled by its σ^32^ (Nonaka et al., 2006). Similarly, we discovered that the *E. chaffeensis* chaperon protein gene (Ech_0471) encoding for DnaK protein is transcribed primarily by σ^32^ (Liu et al., 2013). Genes regulated by σ^32^ are known to induce cellular responses under varieties of stresses confronted during bacterial growth and are likely important for *E. chaffeensis* survival in its hostile host environments and that they may contribute to pathogenicity similar to other Gram negative bacteria (Du et al., 2005; Delory et al., 2006; Slamti et al., 2007; Matsui et al., 2008; Spector and Kenyon, 2012). For example, DnaK in *Vibrio cholerae*, the gene expression regulated by its σ^32^, is involved in causing virulence in a host (Sahu et al., 1994; Chakrabarti et al., 1999; Slamti et al., 2007).

Prior research on *E. coli* (a γ-proteobacteria) provides abundant knowledge regarding gene regulation from its σ^32^- and σ^70^-bound RNAP, while such knowledge for both σ^32^-and σ^70^ bound RNAP in other Gram-negative bacteria, particularly for α-proteobacteria, including for pathogenic organisms is very limited. Importantly, it is unclear how intracellular pathogens, such as *E. chaffeensis*, regulate gene expression to overcome the host stress and adapt to host environmental changes within its arthropod (tick) and vertebrate hosts. To extend knowledge on how *E. chaffeensis* regulates its gene expression, we continue investigations in defining the functions of its RNAP holoenzyme comprising σ^32^ or σ^70^. Transcription derived by an RNAP typically implicates in recognizing and binding to DNA sequence motifs of a promoter; −10 and −35 regions, and the spacer sequences located between the two motifs of a gene promoter (Gross et al., 1998; Paget and Helmann, 2003).

In the current study, we described the mapping of *dnaK* gene promoter recognized primarily by the *E. chaffeensis* RNAP containing σ^32^ using the previously developed *E. coli* surrogate system in the strain, CAG57101 (Koo et al., 2009a; Liu et al., 2013). In *E. coli* CAG57101, its endogenous *rpoH* gene (encoding for σ^32^) is inactivated (Koo et al., 2009a) and in its place, we expressed the *E. chaffeensis* σ^32^ from a plasmid in defining *dnaK* promoter mapping, as direct gene mapping studies are not possible in this and other related intracellular rickettsials. We also investigated the functional domains of *E. chaffeensis* σ^32^ likely important for the RNAP function and in its interactions with the −10 motif and the spacer sequence of *dnaK*.

## Materials and Methods

### *E. coli* Strains and Plasmids

*Escherichia coli* strains used in this study were TOP10 (Invitrogen Technologies, Carlsbad, CA, United States), BL21(DE3) (Novagen, San Diego, CA, United States), and CAG57101 (Koo et al., 2009a). Several plasmid constructs used in this study were obtained from commercial sources or modified from one or more of the existing plasmids. They include the derivatives of pSAKT-Eco_rpoH (previously known as pSAKT32) (Wang and deHaseth, 2003; Koo et al., 2009a), pQF50K-Ech_dnaK (Liu et al., 2013) and pMT504 (Tan and Engel, 1996). Genetic makeup of plasmids described in this study were included in Supplementary Table S1, except those obtained from a commercial source. The plasmid pSAKT-Eco_rpoH containing a p15A origin of replication and an ampicillin resistance gene has *E. coli rpoH* gene under the control of IPTG inducible P_lac_ promoter (Wang and deHaseth, 2003; Koo et al., 2009a). The *E. coli rpoH* from this plasmid was removed by using Q5 Site-Directed Mutagenesis Kit (New England Biolab, Inc., Ipswich, MA, United States) and the modified plasmid was referred as the pSAKT. The pSAKT-Ech_rpoH (previously known as pSAKT32- *Ech_rpoH*) contained *E. chaffeensis rpoH* (Liu et al., 2013). *E. chaffeensis rpoH* variants with substitutions within the 2.3, 2.4, and 3.0 regions of σ^32^ were constructed by mutagenesis using Q5 Site-Directed Mutagenesis Kit (New England Biolab, Inc., Ipswich, MA, United States). The names of the modified pSAKT-Ech_rpoH are provided in Supplementary Table S1.

The pQF50K-Ech_dnaK plasmid and the pQF50K-Ech_dnaK with deletion of −35 motif, which contains the β-galactosidase coding sequence (*lacZ*) with a pMB1 origin of replication and with a kanamycin resistance gene, was reported earlier (Liu et al., 2013). The modified plasmid with deletion of −10 motif was generated from pQF50K-Ech_dnaK plasmid using Q5 Site-Directed Mutagenesis Kit (New England Biolab, Inc., Ipswich, MA, United States). Site directed mutagenesis constructs with mutations at every nucleotide of the −10 motif were also generated from the pQF50K-Ech_dnaK plasmid using Q5 Site-Directed Mutagenesis Kit. The mutants to modify the AT rich spacer sequence of the dnaK promoter were generated similarly by modifying the pQF50K-Ech_dnaK plasmid. The expression plasmids of *E. chaffeensis* wildtype σ^32^ was constructed and used for preparing purified recombinant proteins of σ^32^ as in early reports (Liu et al., 2013).

For *in vitro* transcription analysis, pMT504-Ech_dnaK as transcription template was prepared and reported earlier (Liu et al., 2013). Constructs with various mutations at −10 motif for the *dnaK* promoter for *in vitro* transcription assays were similarly prepared from this plasmid using Q5 Site-Directed Mutagenesis Kit. The lengths of transcripts for the various promoter segments of *dnaK* gene are 162 nucleotides. Integrity of all cloned segments in the plasmid constructs was confirmed by DNA sequence analysis using CEQ 8000 Genetic Analysis System (Beckman Coulter, Fullerton, CA, United States). The names of all engineered plasmids were listed in Supplementary Table S1. Mutagenic oligonucleotides were described in the Supplementary Table S2.

### *E. coli* Growth Conditions and β-Galactosidase Assays

The *E. coli* strain CAG57101 transformed with the recombinant plasmids were grown as in early reports (Koo et al., 2009a; Liu et al., 2013). Briefly, cultures were grown at 30°C in Luria–Bertani (LB) medium with chloramphenicol (30 μg/ml) and spectinomycin (50 μg/ml) in support of the strain’s growth, and by ampicillin (100 μg/ml) for maintaining the pSAKT-derived plasmids. To assess the functions and impact of various mutations within the promoter regions of genes encoding *dnaK*, pQF50K-derived plasmid containing the promoter segments were also maintained by growing *E. coli* cultures with the addition of kanamycin (50 μg/ml). *E. coli* cultures of CAG57101 in LB medium were grown overnight with appropriate antibiotic supplements which were diluted 1:100 into a fresh medium containing appropriate antibiotics and the growth was continued for 2 h. Subsequently, cultures were then induced with 1 mM IPTG for 3 h before harvesting, when OD at 600 nm reached between 0.6 and 0.8. Lysates were prepared and used to measure β-galactosidase enzyme activity using a β-Gal Assay Kit (Invitrogen Technologies, Carlsbad, CA, United States). All experiments were performed three independent times with independently grown cultures; specific activity of β-galactosidase was calculated as outlined in the kit protocol.

### *In vitro* Transcription Assays

In vitro transcription reactions were performed in 10 μl reaction mixture containing 0.1 picomoles each of the supercoiled plasmid DNA as the template and using RNAP holoenzyme containing recombinant E. chaffeensis σ^32^ (Liu et al., 2013). The holoenzyme was prepared by mixing 0.5 μl of 1:10 diluted stock of *E. coli* core enzyme (Epicentre, Madison, WI, United States) mixed with 10-fold molar excess of purified recombinant E. chaffeensis σ^32^ and kept in ice for 30 min prior to using for the reactions. The transcription reactions were performed at 37°C for 20 min, and the reactions were terminated by adding 7 μl of stop solution (95% formamide, 20 mM EDTA, 0.05% bromophenol blue and 0.05% xylene cyanol). Six microliters each of the samples were resolved on a 6% polyacrylamide sequencing gel with 7 M urea, then gels were transferred to a Whatman paper, dried and 162 nucleotide transcripts were visualized by exposing an X-ray film to the gels. The transcripts were quantified using ImageJ software^1^.

### Bioinformatics

Multiple DNA alignments were done using the programs Clustal X version 2.0 with default parameters (Larkin et al., 2007).

### Statistical Analysis

Statistical analyses were performed using Student’s *t*-test, and a *P*-value < 0.05 was considered significant with a single asterisk.

## Results

### The −10 Motif Is Needed for the *E. chaffeensis dnak* Gene Transcription by RNAP Holoenzyme Constituting of Its σ^32^

**FIGURE 1:**
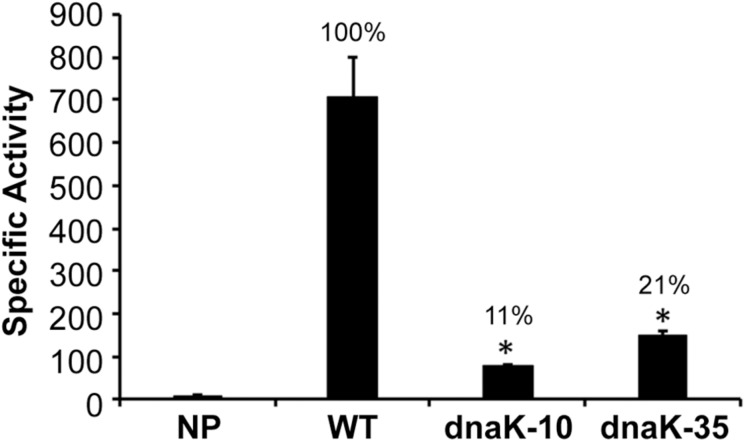
Importance of –10 and –35 motifs of *E. chaffeensis dnaK* gene promoter assessed in *E. coli* CAG57101 expressing *E. chaffeensis* σ^32^. The β-galactosidase expression driven by *E. chaffeensis* wild-type *dnaK* (WT) and the promoter with –35 motif or –10 motif deletions (*dnaK-10* and *dnaK-35*) was assessed relative to no promoter (NP) control. Significant changes in the β-galactosidase were identified compared with the data observed for WT. All values are averages of at least three independent experiments; error bars indicated one standard deviation. ^*^p-value < 0.05.

Our prior studies demonstrated that −35 motif, but not −10 motif, is required for the σ^70^-bound RNAP holoenzyme transcription from σ^70^-dependent promoters in *E. chaffeensis* (Liu et al., 2016). We also reported earlier that −35 motif is similarly required for the *dnaK* gene transcription by *E. chaffeensis* σ^32^–bound RNAP holoenzyme (Liu et al., 2013). To test whether or not the −10 motif of *dnaK* promoter is required for the σ^32^-dependent gene regulation, plasmid constructs lacking −10 or −35 motifs of the promoter were cloned upstream to the β-galactosidase coding sequence in an *E. coli* mutant deficient for its σ^32^ expression that is functionally complemented with the *E. chaffeensis* σ^32^ (Figure 1). The −10 motif deletion and similarly the −35 motif deletion resulted in a significant reduction of β-galactosidase activity (to 11 and 21%, respectively; *p* ≤ 0.0005) compared to the *dnaK* wildtype (WT). The reduction of the β-galactosidase activity for the −10 motif deletion was similar to the negative control where the promoter segment was absent (NP).

### Identifying the Critical Sequence Determinants of −10 Motif of *E. chaffeensis dnaK*

**FIGURE 2:**
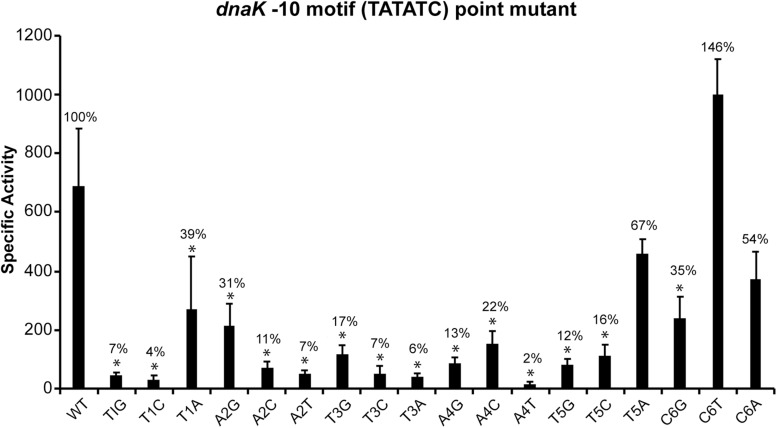
Mapping the sequence determinants of –10 motifs in *E. chaffeensis dnaK* gene promoter. The β-galactosidase expression driven by *E. chaffeensis* promoter constructs containing point mutations at each of the six nucleotide positions of the –10 motifs of *dnaK* were measured in CAG57101 expressing *E. chaffeensis* σ^32^. The experiment included the wildtype promoter control (WT). Each mutation is identified with a change of the nucleotide at each position to the modified nucleotide. β-galactosidase expression was presented relative to WT control. All values are averages of at least three independent experiments; error bars indicated one standard deviation. ^*^p-value < 0.05.

The consensus sequence of −35 motif for *E. chaffeensis* promoters recognized by its σ^32^ (TTGTAT) is similar to the consensus −35 motif of σ^32^-dependent promoters in *E. coli* (TTGAAA) and similarly it shares extensive homology to −35 motif for the genes recognized by σ^70^ (TTGNTT) (Nonaka et al., 2006; Liu et al., 2013). The consensus −10 motif of *E. chaffeensis* promoters recognized by its σ^32^ (TATATN) is also similar to its consensus −10 motif recognized by its σ^70^ (TATTNT), however, it differs significantly from the consensus −10 motif of *E. coli* σ^32^-dependent promoters (CCCCATWT) (Nonaka et al., 2006; Koo et al., 2009a; Liu et al., 2013). While deletion of −10 motif from σ^70^-dependent promoters has no impact on promoter activities in *E. chaffeensis* (Liu et al., 2016), such deletion from the σ^32^-dependent *dnaK* resulted in significant and 90% reduction in the promoter activity (Figure 1). These novel data suggest that, contrary to σ^70^-bound RNAP (Liu et al., 2016), the −10 motif plays a critical role for σ^32^-bound RNAP in *E. chaffeensis.* We therefore performed detailed point mutation experiments to define the critical sequence determinants of the −10 motif for the *dnaK* promoter activity (Figure 2). We have made substitution mutations at each base of the six-nucleotide motif (TATATC) and evaluated the impact of each mutation by measuring changes in β-galactosidase expression in CAG57101 *E. coli* functionally complemented with *E. chaffeensis* σ^32^. A specific substitution mutation was indicated by combination of letters and numbers. For example, T1A indicates a change from T to A transversion at the first position in the −10 motif. One or more substitutions at all six nucleotide positions resulted in significant decline in the promoter activity of *dnaK*. Substitutions at the first five nucleotides to any other nucleotide resulted in significant promoter activity drop. Substitutions in the first position from T1G or T1C resulted in the near complete loss of promoter activity, while T1A resulted in the reduction of promoter activity to 39%. Similarly, in the second position, A2C and A2T mutations caused significant loss of promoter activity (reduced to 17% and 7% compared to the wildtype), whereas A2G mutation caused decline of two thirds of activity similar to T1A substitution. In the third and fourth positions, changes to any other nucleotide had the greatest impact in promoter activity decline (78–98% reduction). Mutations in the fifth position to T5G or T5C had a greater impact (a near 90% decline), while reduction was less apparent for the T5A that is similar to the mutations in the first position T. In the sixth position, only C6G mutation resulted in significant decline in the promoter activity to 35%.

### *In vitro* Transcription Assays to Verify Sequence Determinants of −10 Motif Mapped in *E. coli* CAG57101

**FIGURE 3:**
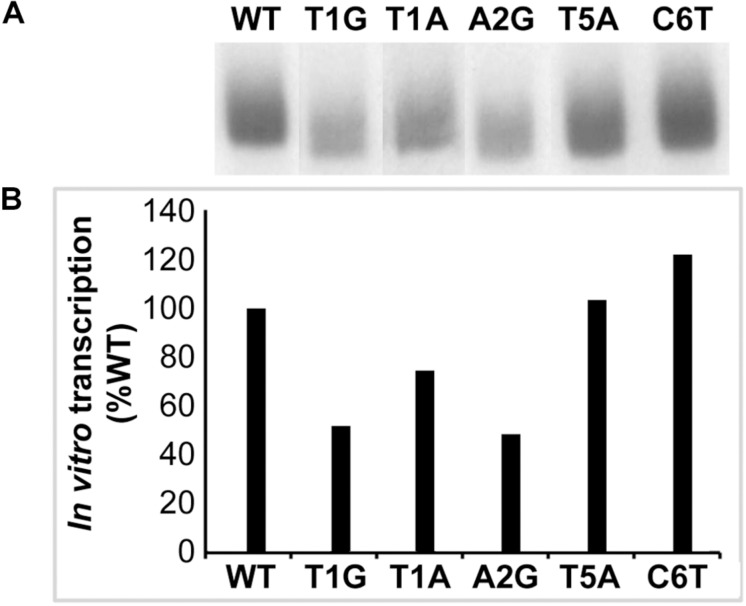
*In vitro* transcription analysis validating the *E. chaffeensis* gene promoter mutants spanning the –10 motifs. Five randomly selected mutations at –10 motifs of *dnaK* were examined by *in vitro* transcription assays using RNAP holoenzyme containing *E. chaffeensis* recombinant σ^32^. The mutants of promoter segments cloned upstream to the G-less cassette in the pMT504 plasmid were used in the assays. The abundance of transcripts was captured as ^32^P incorporation. Intensity of a band signals in a gel for *in vitro* transcripts made for the wild-type and mutant *dnaK* were assessed using the software ImageJ (https://imagej.nih.gov/ij/). Panel **(A)** has the image data and panel **(B)** included the quantitative data collected from the image signals. The bars show the relative transcription products of mutant promoters as the percentage of transcripts compared to the wild-type promoter for *E. chaffeensis* recombinant σ^32^. (WT and various mutant promoter constructs are identified as in Figure 2).

To validate the results in defining the −10 motif in the *E. coli* surrogate system, we performed *in vitro* transcription assays using several randomly selected promoter mutation constructs; the assays were performed using RNAP holoenzyme reconstituted with the recombinant *E. chaffeensis* σ^32^. We selected five *dnaK* −10 motif mutants for this experiment and compared the results with the wildtype promoter. Both wildtype and mutated versions of *dnaK* promoter segments were cloned into the G-less cassette and used as templates in the *in vitro* transcription assays (Figures 3A,B). Consistent with the results recorded with the *E. coli* CAG57101 system, mutants TIA, T1G and A2G produced lesser transcripts compared to the wildtype *dnaK.* There was no significant difference for the T5A mutation compared to the wildtype, which is also consistent with the results observed in the *E. coli* system. In the sixth position, C6T mutation caused an increase of *in vitro* transcript level, which is also similar to the enhanced promoter activity observed in the *E. coli* CAG57101experiments.

### The Spacer Sequences Affect Promoter Activity

**FIGURE 4:**
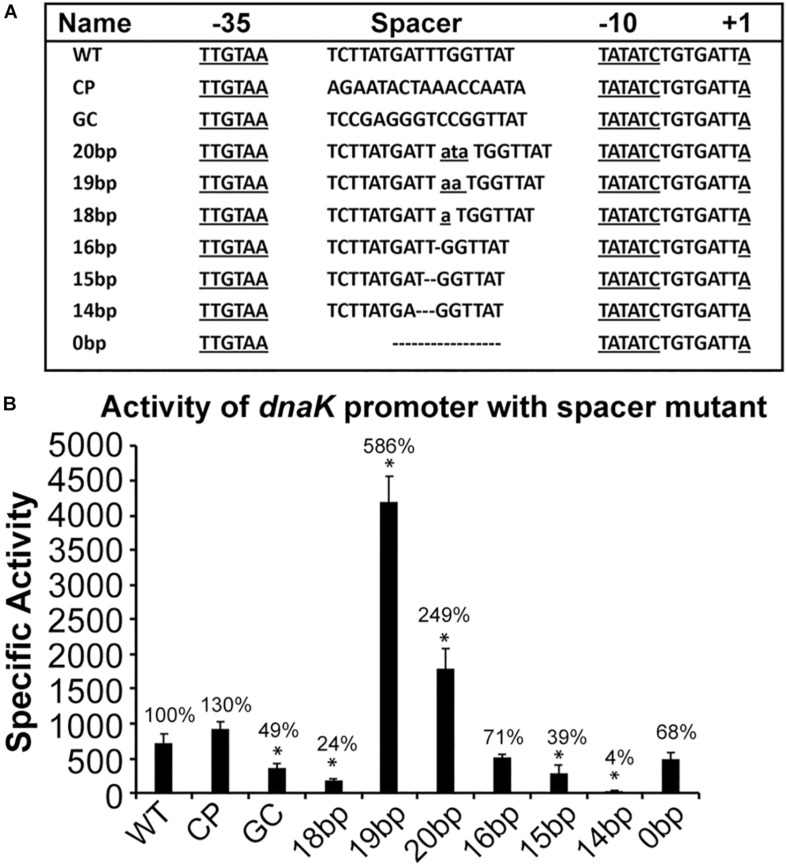
AT-rich spacer sequence located between –10 and –35 motif contributes to altering the promoter activity of *Ehrlichia chaffeensis dnaK* gene. Promoter fragments used in the assays are as in Supplementary Figure S1 for wild-type *dnaK*. **(A)** Sequence spanning from +1 to –35 motif and the AT-rich spacer sequence is presented for the wild-type construct (WT) and for the constructs with modified spacer sequences which included replacing the AT-rich spacer with complementary sequence (CP), with GC rich spacer sequence (GC), changing the size of spacer from 17 bp (WT) to 20 bp (20bp), 19 bp (19bp), 18 bp (18bp), 16 bp (16bp), 15 bp (15bp), and 14 bp (14bp) and deleting entire 17 bp spacer (0bp). Lower case with underline indicated inserted nucleotides in spacer sequence and the break line refers to the deletion nucleotides from WT *dnaK* promoter. **(B)** The β-galactosidase activity was driven by promoters of WT, CP, GC, 20bp, 19bp, 18bp, 16bp, 15bp, 14bp, and 0bp in *E. coli* (CAG57101) with expressing *E. chaffeensis* σ^32^. All values are averages of at least three independent experiments; error bars indicated one standard deviation. ^*^p-value < 0.05.

Previous studies in *E. coli* demonstrate that spacer sequences located between −10 and −35 motifs contribute to promoter activities (Aoyama et al., 1983; Mulligan et al., 1985; Hook-Barnard and Hinton, 2007, 2009; Singh et al., 2011). In particular, nucleotides present in the spacer sequence and its length play critical role for a promoter activity. Further, a short C-rich region upstream to −10 motif in *E. coli* and in other γ-proteobacteria is identified as important extended −10 motif required for efficient transcription by σ^32^-containing RNAP holoenzyme (Nonaka et al., 2006; Slamti et al., 2007; Koo et al., 2009a; Stoll et al., 2009), while such C-rich sequence does not exist in *E. chaffeensis* gene promoters, including in the *dnaK* promoter. Our previous studies for σ^70^-dependent promoter genes suggested that changes to the spacer sequence impact a promoter activity (Liu et al., 2016). We, therefore, investigated the importance of *dnaK* spacer sequence, including in determining about how the lack of C-rich sequence impact the promoter activity. Nine spacer mutants were prepared where nucleotides within the spacer sequence were modified; they included replacing the spacer sequence with its complementary sequence (CP), or with a high GC content spacer while keeping the spacer length constant (GC), or by increasing the spacer sequence lengths from 17 bp to 18, 19, or 20 bps or by decreasing it to 16, 15, or 14 bp and finally by deleting the spacer sequence completely. These different spacer mutant constructs were depicted in Figure 4A. The β-galactosidase expression was then assessed for all these modified spacer promoter segments and compared to wildtype (WT) promoter construct in the *E. coli* surrogate system (CAG57101) (Figure 4B). The CP mutant caused a minor, non-significant increase in the promoter activity (30% increase). The spacer substitution with GC resulted in a significant 50% decline in the β-galactosidase expression. Increasing the spacer length to 18 bp or decreasing to 14 or 15 bp caused a major decline in the promoter activity, although the greatest decline was observed with the 14 bp spacer (96% drop), while reducing the length to 16 bp had no impact. Increasing the spacer lengths to 19 or 20 bp resulted in much higher enhancement of the promoter activity (586% and 249%, respectively). Deletion of the entire spacer sequence had no impact on the promoter activity compared to the WT promoter. We detected the presence of another 15 bp spacer like sequence and an alternative −35 motif sequence in the complete deletion spacer construct; thus, it is highly likely that these sequences served as alternate spacer and −35 motif for the RNAP (Supplementary Figure S1).

### Substitution Mutations in *E. chaffeensis* σ^32^

**FIGURE 5:**
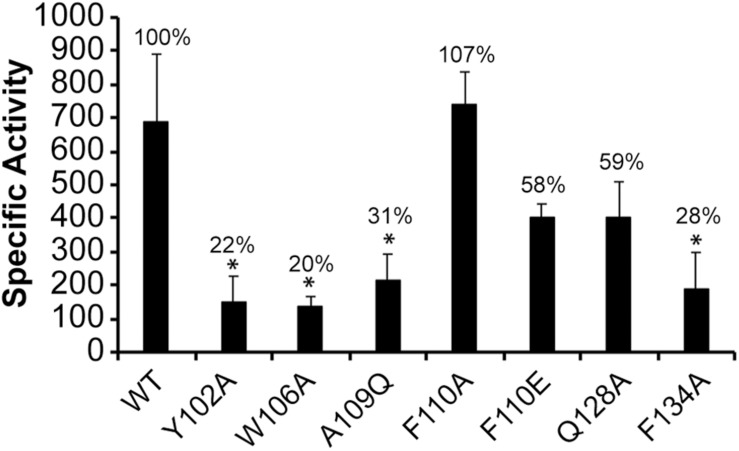
Substitutions at six amino acids located in regions 2.3, 2.4, and 3.0 of *E. chaffeensis* σ^32^ impacting the promoter activity of the wild-type *E. chaffeensis dnaK*. Mutations at six residues (Y102, W106, A109, F110, Q128, and F134) of *E. chaffeensis* σ^32^ were assessed with the wild-type *dnaK* promoter. β-galactosidase expression was measured for the mutant proteins relative to the wild-type (WT) *E. chaffeensis* σ^32^ in CAG57101. All values are averages of at least three independent experiments; error bars indicated one standard deviation. ^*^p-value < 0.05.

Prior studies in *E. coli* revealed that the −10 motif of σ^32^-dependent promoters is recognized by few amino acids within the regions 2.3, 2.4, and 3.0 of σ^32^ protein (Kourennaia et al., 2005; Koo et al., 2009a). To determine what amino acids spanning these regions of *E. chaffeensis* σ^32^ would contribute to the promoter activity, we have made substitution mutations at 6 different amino acid positions likely alter the functional domains of the pathogen σ^32^. The amino acids for substitution mutations were selected based on their homology with the *E. coli* σ^32^ (Supplementary Figure S2). Tryptophan (W) 108 of *E. coli* σ^32^ within the region 2.4 is identified as important for recognition of −13C in a promoter recognized by it (Kourennaia et al., 2005; Koo et al., 2009a). In *E. chaffeensis* σ^32^, W is located at 106. Glutamic acid (E) at position 112 in *E. coli* σ^32^ is also implicated for its contribution to −13C recognition and interaction with its σ^32^-dependent promoters (Koo et al., 2009a). Phenylalanine (F) at position 110 of *E. chaffeensis* σ^32^ is in the homologous position to E112 in *E. coli* σ^32^. As *E. chaffeensis dnaK* lacks C-rich region upstream to its −10 motif, we expected that amino acid substitution mutations at these two positions in *E. chaffeensis* σ^32^ would not have any impact. Lysine (K) at position 130 in region 3.0 of *E. coli* σ^32^ is similarly implicated for the recognition of the C-rich region for the σ^32^-promoters; *groE* and *grpE* (Koo et al., 2009a). Glutamin (Q) at position 128 in region 3.0 of *E. chaffeensis* σ^32^ is the amino acid at the homologous position to K130 of *E. coli* σ^32^. Substitution of W106 to A caused significant reduction of the β-galactosidase expression (80% decline), while F110 substitution to A or E (alanine or glutamic acid) in *E. chaffeensis* σ^32^ did not significantly alter the promoter function (Figure 5). Similarly, substitution of Q128 to A did not significantly impact *E. chaffeensis dnaK* promoter activity. The mutational data with the exception of W106 are consistent with the lack of C-rich region in *E. chaffeensis.* However, W106 may be critical for the promoter activity independent of the C-rich sequence, at least in *E. chaffeensis*. Phenylalanine (F) at position 104 within the 2.3 region of *E. coli* σ^32^ is identified as critical for its structural integrity and activity of σ^32^ (Kourennaia et al., 2005). In *E. chaffeensis* σ^32^, a polar amino acid (at position 102) {tyrosine (Y)} is present at the position homologous to F104. Similarly, A111 of *E. coli* σ^32^ is implicated for its binding to core RNAP (Kourennaia et al., 2005). Y102 to A caused a significant decline of *E. chaffeensis* σ^32^ activity (88% reduction). Likewise, substitution of A109 to glutamine (Q) in *E. chaffeensis* σ^32^ that is homologous to A111of *E. coli* σ^32^ resulted in a significant decline of activity (about 31% of WT level). Previous studies in *E. coli* demonstrate that substitution of F136A within the region 3.0 reduces the interaction between core RNAP and σ^32^ thus leading to 80% decline in promoter activity for its *groE* gene (Joo et al., 1997; Kourennaia et al., 2005; Koo et al., 2009a). A similar substitution in *E. chaffeensis* σ^32^; F134A also caused similar decline of its activity for the *dnaK* promoter (reduced to 28% activity compared to WT *E. chaffeensis* σ^32^). Together, *E. chaffeensis* σ^32^ substitution mutation experiments allowed the identification of critical functional domains engaged in σ^32^-bound RNAP interactions with the *dnaK* promoter, including in confirming that the C-rich region is not critical for its function.

### Differences in the −10 Motifs Between *E. coli* and *E. chaffeensis* Are Sufficient in Having Differential σ^32^ Functions

**FIGURE 6:**
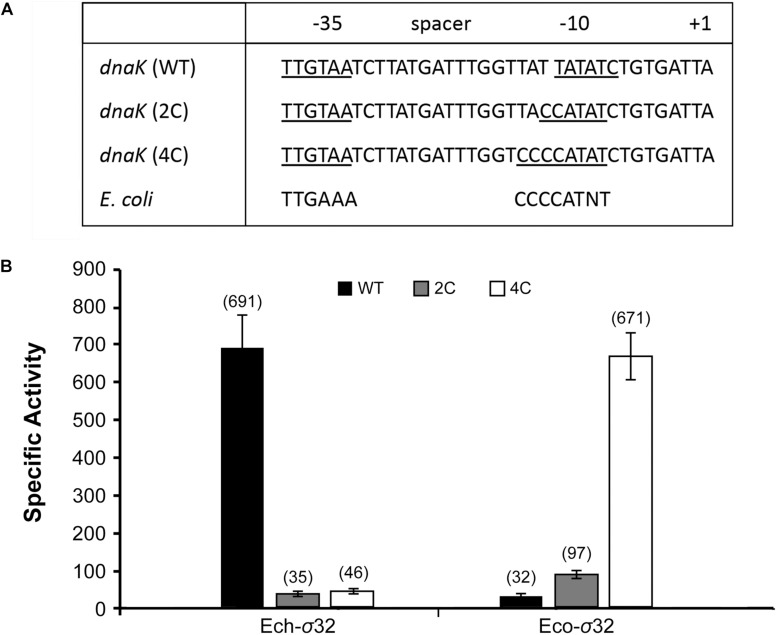
Sequence variations in –10 motif and its immediate upstream sequence define specificities for *E. coli-* and *E. chaffeensis* σ^32^. The –10 motif for wild-type *dnaK* promoter (WT) and for the modified sequences where TT and TATT near –10 motif are modified to CC (2C) and CCCC (4C), respectively (panel **A**). The inclusion of 2C and 4C make the –10 motif of *dnaK* promoter to be similar to consensus sequence of –10 motif for *E. coli*. *E. chaffeensis* σ^32^ (Ech-σ^32^) and *E. coli* σ^32^ (Eco- σ^32^) recognized the WT, 2C, and 4C promoters differently (panel **B**). β-galactosidase expression as specific activity values were in parenthesis. All values are averages of at least three independent experiments; error bars indicated one standard deviation.

Unlike *E. coli* and other γ- proteobacterial gene promoters, −10 motif of *E. chaffeensis* σ^32^-dependent *dnaK* promoter (TATATN) is distinct in lacking a C-rich sequence upstream to −10 motif (Nonaka et al., 2006; Koo et al., 2009a; Liu et al., 2013). Results presented in the previous section suggest that the C-rich region is indeed not required for *E. chaffeensis* σ^32^ dependent *dnaK* promoter function. To further map how the variations in −10 motif and spacer sequence in *E. chaffeensis dnaK* make it unique for this intracellular pathogen promoter function, we prepared two modified constructs where two or four nucleotides spanning between the spacer sequence and −10 motif (TT or TATT, respectively) were replaced with either two Cs or four Cs (Figure 6A). Importantly, these sequence modifications change the *E. chaffeensis* −10 motif to be more similar to the *E. coli* consensus −10 motif (CCCCATWT) (Figure 6A). The modified constructs having 2Cs and 4Cs were then assessed in the *E. coli* surrogate system expressing either *E. chaffeensis* σ^32^ (Ech- σ^32^) or *E. coli* σ^32^ (Eco-σ^32^). Compared to the wildtype *dnaK* promoter, both the 2C and 4C mutants caused drastic decline in the β-galactosidase expression by as much as 15-fold for *E. chaffeensis* σ^32^ (Figure 6B). On the contrary, while wildtype *dnaK* promoter had a minimal β-galactosidase expression with the *E. coli* σ^32^, the 2Cs and 4Cs substitutions caused a significant and a step-wise increase of β-galactosidase expression to 2.8-fold and 14.5-fold, respectively. Together, these results suggest that the inclusion of C-rich sequences is sufficient in altering the promoter specificities of *E. chaffeensis* to be similar to *E. coli* σ^32^-dependent RNAP.

## Discussion

Because obligate intracellular bacteria reside within the cytosol or a phagosome of an infected host cell, they encounter minimal environmental changes, possibly compared to free-living bacteria, such as *E. coli*. Obligate intracellular bacteria typically have reduced genomes and consequently their genomes also have limited numbers of sigma factors. For example, *E. chaffeensis* genome has only two sigma factor genes encoding for a constitutive and an alternative sigma factor, σ^70^ and σ^32^, respectively (Dunning Hotopp et al., 2006). On the contrary, a non-pathogenic bacterium, such as *E. coli*, contains seven sigma factors, including σ^70^ and σ^32^ (Gruber and Gross, 2003; Tripathi et al., 2014). Alternate sigma factor; σ^32^ is known to regulate genes involved in overcoming environmental stresses (Zhao et al., 2005; Martínez-Salazar et al., 2009). Prior studies in *E. coli, V. cholerae*, *Neisseria gonorrhoeae*, and *Francisella tularensis* suggest that σ^32^ regulon involves several heat-shock proteins, also known as chaperon proteins; Hsp40, HspG, Dnak, DnaJ, GroES, and GroEL. These proteins are involved in temperature-induced stress control of the organisms (Zhao et al., 2005; Gunesekere et al., 2006; Nonaka et al., 2006; Slamti et al., 2007). These proteins play critical roles in both preventing denaturation of proteins and also to promote renaturation of aggregated proteins (Gragerov et al., 1992; Nishihara et al., 1998; Mogk et al., 1999). Some of the proteins involved in regulating stress response, such as ClpB, HspG, and DnaK, are also considered important for the intracellular survival of a pathogenic bacterium, *F. tularensis* (Tempel et al., 2006; Su et al., 2007; Weiss et al., 2007; Meibom et al., 2008). The sigma factor, σ^32^, is identified as critical for promoting transcription of genes engaged in overcoming stressful environments for bacteria to promote the reduction of accumulation of misfolded and aggregated proteins (Gragerov et al., 1992; Slamti et al., 2007; Guisbert et al., 2008).

*Ehrlichia chaffeensis* and related *Anaplasmataceae* family pathogens have the ability adapt to vertebrate and tick hosts. However, it is unclear how tick transmitted pathogens regulate gene expression in tick and vertebrate hosts. Studying gene regulation will aid in understanding how rickettsial pathogens adapt to dual hosts and sense nutrient, starving, temperature, and other stressful environments within an infected host cell. Previous studies revealed that *E. chaffeensis* has two morphological forms, dense core cell as the pathogen’s infectious form and reticulate cell, which replicates within a phagosome of an infected host cell (Zhang et al., 2007; Dedonder et al., 2012). We recently reported that a stress response protein, ClpB, transcripts are higher during the replicative stage of the pathogen, while gene expression of another heat shock protein, DnaK, and the stress response sigma factor, RpoH, remained as constitutively expressed throughout the replicating stage (Liu et al., 2013; Zhang et al., 2013). Further, we also reported that *E. chaffeensis* sigma factors, σ^32^ and σ^70^, function cooperatively in transcribing pathogen genes, but with varying affinities (Liu et al., 2013). The *dnaK* has higher affinity for RNAP containing *E. chaffeensis* σ^32^ compared to σ^70^ (Liu et al., 2013). The current study is the first in defining *E. chaffeensis* σ^32^-dependent gene promoter region of *dnaK*.

Despite our recent advances in establishing tractable genetics for *E. chaffeensis* (Wang et al., 2017), studying gene regulation remains a challenge due to lack of a well-established methods to maintain extracellular plasmids, as the pathogen and other related *Anaplasmataceae* pathogens lack natural plasmids. To overcome this challenge, in the current study, we utilized the *E. coli* surrogate system to map promoter-binding domains required for gene expression in *E. chaffeensis* for a σ^32^-dependent gene promoter. We validated the results from the *E. coli* surrogate system using *in vitro* transcription assays which we developed earlier (Liu et al., 2013, 2016). *In vitro* transcription system is applied well in understanding intracellular bacterial gene regulation for pathogens, such as *Chlamydia trachomatis*, for which a tractable genetic system is equally not well developed, which can support gene regulation studies (Mathews et al., 1993; Tan and Engel, 1996; Tan et al., 1998; Yu and Tan, 2003; Shen et al., 2004; Akers and Tan, 2006; Rao et al., 2009; Bao et al., 2011, 2012). Hence, in view of the technical challenges, methods described in the current study are innovative in mapping *E. chaffeensis* gene regulation, and that the study will lead the way for similar investigations in other related *Anaplasmataceae* family pathogens.

RNA polymerase holoenzyme containing σ^70^ of *E. coli* transcribes housekeeping genes by recognizing two highly conserved motifs of a gene promoter; referred as −10 and −35 motifs (Gross et al., 1998). We reported earlier that the consensus −10 and −35 motifs for *E. chaffeensis* σ^70^-dependent promoters (TATTNT and TTGNTT, respectively) (Liu et al., 2013) are similar to *E. coli* consensus −10 and −35 (TATAAT and TTGACA, respectively) (Singh et al., 2011; Shimada et al., 2014). We reported that the −35 motif and the AT-rich spacer sequences are important for a gene-specific regulation by σ^70^-dependent promoters (Liu et al., 2016). The predicted *E. chaffeensis* consensus −35 motif for the alternative sigma factor (σ^32^) is also similar to *E. coli* consensus −35 motif (Nonaka et al., 2006; Koo et al., 2009a,b; Liu et al., 2013). The −10 motif of *E. chaffeensis* σ^32^-dependent promoters (TATATN), however, differs substantially from the consensus *E. coli*−10 motif (CCCCATNT) (Nonaka et al., 2006; Koo et al., 2009a,b; Liu et al., 2013). The consensus −35 motif is also extensively conserved among σ^32^-dependent promoters in proteobacteria (Supplementary Table S3), while −10 motif of σ^32^-dependent promoters vary considerably among various classes of proteobacteria. For example, many γ-proteobacteria contain a C-rich sequence upstream to the −10 motif and is implicated in regulating σ^32^-dependent gene regulation (Nonaka et al., 2006; Slamti et al., 2007; Grall et al., 2009; Stoll et al., 2009). Such sequence, however, is absent in *E. chaffeensis* σ^32^-dependent gene promoters (Liu et al., 2013). Thus, we hypothesized that the pathogen is evolved to possess a distinctive −10 motif and that it plays a critical role in σ^32^-dependent promoter regulation. Previous studies suggest that the length of a spacer sequence between the −10 and −35 motifs and the composition of specific nucleotides present within a spacer affect promoter activity (Aoyama et al., 1983; Mulligan et al., 1985; Hook-Barnard and Hinton, 2009; Singh et al., 2011; Liu et al., 2016). In particular, spacer sequences influence the three-dimensional structure of a promoter and any modifications to a spacer sequence, therefore, affect the efficiency of interactions between the RNAP holoenzyme with a promoter sequence either positively or negatively (Rud et al., 2006; Hook-Barnard and Hinton, 2009; Singh et al., 2011). Consistent with prior studies, our current investigation similarly revealed the importance of spacer sequence in contributing to the promoter activity for the *E. chaffeensis dnaK* gene transcribed by its σ^32^. We previously reported that modifications to spacer sequences similarly impact *E. chaffeensis* genes transcribed by σ^70^ genes and that changes to spacer sequences alter the curvature of a promoter region (Liu et al., 2016).

In summary, we mapped *E. chaffeensis dnaK*−10 and −35 motifs and the spacer sequence upstream to it by performing detailed mutational analysis. Furthermore, this study confirmed that the C-rich region–specific interactions between a gene promoter and σ^32^ protein domains, known to be critical for *E. coli*, are absent in *E. chaffeensis*, which makes the pathogen gene regulation distinct, as judged from characterizing the *dnaK* gene promoter.

## Data Availability

The datasets generated for this study are available on request to the corresponding author.

## Author Contributions

RG conceived and directed the research design. HL contributed to the research plan and performed the experiments. HL and RG evaluated the data and prepared the manuscript.

## Conflict of Interest Statement

The authors declare that the research was conducted in the absence of any commercial or financial relationships that could be construed as a potential conflict of interest.

## Footnotes

1 http://rsb.info.nih.gov/ij

## References

[B1] Akers J. C. Tan M. (2006). Molecular mechanism of tryptophan-dependent transcriptional regulation in *Chlamydia trachomatis*. *J. Bacteriol.* 188 4236–4243. 10.1128/jb.01660-05 16740930 PMC1482941

[B2] Aoyama T. Takanami M. Ohtsuka E. Taniyama Y. Marumoto R. Sato H. (1983). Essential structure of *E. coli* promoter: effect of spacer length between the two consensus sequences on promoter function. *Nucleic Acids Res.* 11 5855–5864. 10.1093/nar/11.17.5855 6310517 PMC326322

[B3] Bao X. Nickels B. E. Fan H. (2012). *Chlamydia trachomatis* protein GrgA activates transcription by contacting the nonconserved region of sigma66. *Proc. Natl. Acad. Sci. U.S.A.* 109 16870–16875. 10.1073/pnas.1207300109 23027952 PMC3479454

[B4] Bao X. Pachikara N. D. Oey C. B. Balakrishnan A. Westblade L. F. Tan M. (2011). Non-coding nucleotides and amino acids near the active site regulate peptide deformylase expression and inhibitor susceptibility in *Chlamydia trachomatis*. *Microbiology* 157 2569–2581. 10.1099/mic.0.049668-0 21719536 PMC3352175

[B5] Breitschwerdt E. B. Hegarty B. C. Hancock S. I. (1998). Sequential evaluation of dogs naturally infected with *Ehrlichia canis*, *Ehrlichia chaffeensis*, *Ehrlichia equi*, *Ehrlichia ewingii*, or *Bartonella vinsonii*. *J. Clin. Microbiol.* 36 2645–2651. 9705408 10.1128/jcm.36.9.2645-2651.1998 PMC105178

[B6] Browning D. F. Busby S. J. W. (2016). Local and global regulation of transcription initiation in bacteria. *Nat. Rev. Microbiol.* 14:638. 10.1038/nrmicro.2016.103 27498839

[B7] Chakrabarti S. Sengupta N. Chowdhury R. (1999). Role of DnaK in *in vitro* and *in vivo* expression of virulence factors of *Vibrio cholerae*. *Infect Immun.* 67 1025–1033. 10024539 10.1128/iai.67.3.1025-1033.1999 PMC96425

[B8] Chamberlin M. Kingston R. Gilman M. Wiggs J. Devera A. (1983). Isolation of bacterial and bacteriophage RNA polymerases and their use in synthesis of RNA *in vitro*. *Methods Enzymol.* 101 540–568. 10.1016/0076-6879(83)01037-x 6350819

[B9] Cheng C. Nair A. D. Indukuri V. V. Gong S. Felsheim R. F. Jaworski D. (2013). Targeted and random mutagenesis of *Ehrlichia chaffeensis* for the identification of genes required for *in vivo* infection. *PLoS Pathog.* 9:e1003171. 10.1371/journal.ppat.1003171 23459099 PMC3573109

[B10] Crosby F. L. Wamsley H. L. Pate M. G. Lundgren A. M. Noh S. M. Munderloh U. G. (2014). Knockout of an outer membrane protein operon of *Anaplasma marginale* by transposon mutagenesis. *BMC Genomics* 15:278. 10.1186/1471-2164-15-278 24725301 PMC4198910

[B11] Darby A. C. Cho N.-H. Fuxelius H.-H. Westberg J. Andersson S. G. E. (2007). Intracellular pathogens go extreme: genome evolution in the Rickettsiales. *Trends Genet.* 23 511–520. 10.1016/j.tig.2007.08.002 17822801

[B12] Davidson W. R. Lockhart J. M. Stallknecht D. E. Howerth E. W. Dawson J. E. Rechav Y. (2001). Persistent *Ehrlichia chaffeensis* infection in white-tailed deer. *J. Wildl. Dis.* 37 538–546. 11504227 10.7589/0090-3558-37.3.538

[B13] Dawson J. E. Biggie K. L. Warner C. K. Cookson K. Jenkins S. Levine J. F. (1996). Polymerase chain reaction evidence of *Ehrlichia chaffeensis*, an etiologic agent of human monocytic ehrlichiosis, in dogs, from southeast Virginia. *Am. J. Vet. Res.* 57 1175–1179. 8836370

[B14] Dedonder S. E. Cheng C. Willard L. H. Boyle D. L. Ganta R. R. (2012). Transmission electron microscopy reveals distinct macrophage- and tick cell-specific morphological stages of *Ehrlichia chaffeensis*. *PLoS One* 7:e36749. 10.1371/journal.pone.0036749 22615806 PMC3352939

[B15] Delory M. Hallez R. Letesson J. J. De Bolle X. (2006). An RpoH-like heat shock sigma factor is involved in stress response and virulence in *Brucella melitensis* 16M. *J. Bacteriol.* 188 7707–7710. 10.1128/jb.00644-06 16936018 PMC1636281

[B16] Du Y. Lenz J. Arvidson C. G. (2005). Global gene expression and the role of sigma factors in *Neisseria gonorrhoeae* in interactions with epithelial cells. *Infect. Immun.* 73 4834–4845. 10.1128/iai.73.8.4834-4845.2005 16040997 PMC1201249

[B17] Dugan V. G. Little S. E. Stallknecht D. E. Beall A. D. (2000). Natural infection of domestic goats with *Ehrlichia chaffeensis*. *J. Clin. Microbiol.* 38 448–449. 10618139 10.1128/jcm.38.1.448-449.2000 PMC88747

[B18] Dumler J. S. Sutker W. L. Walker D. H. (1993). Persistent infection with *Ehrlichia chaffeensis*. *Clin. Infect. Dis.* 17 903–905. 10.1093/clinids/17.5.903 8286638

[B19] Dunning Hotopp J. C. Lin M. Madupu R. Crabtree J. Angiuoli S. V. Eisen J. (2006). Comparative genomics of emerging human *ehrlichiosis* agents. *PLoS Genet.* 2:e21. 10.1371/journal.pgen.0020021 16482227 PMC1366493

[B20] Faburay B. Liu H. Peddireddi L. Ganta R. R. (2011). Isolation and characterization of *Ehrlichia chaffeensis* RNA polymerase and its use in evaluating p28 outer membrane protein gene promoters. *BMC Microbiol.* 11:83. 10.1186/1471-2180-11-83 21513529 PMC3108270

[B21] Felsheim R. F. Herron M. J. Nelson C. M. Burkhardt N. Y. Barbet A. F. Kurtti T. J. (2006). Transformation of *Anaplasma phagocytophilum*. *BMC Biotechnol.* 6:42. 10.1186/1472-6750-6-42 17076894 PMC1635035

[B22] Gragerov A. Nudler E. Komissarova N. Gaitanaris G. A. Gottesman M. E. Nikiforov V. (1992). Cooperation of GroEL/GroES and DnaK/DnaJ heat shock proteins in preventing protein misfolding in *Escherichia coli*. *Proc. Natl. Acad. Sci. U.S.A.* 89 10341–10344. 10.1073/pnas.89.21.10341 1359538 PMC50334

[B23] Grall N. Livny J. Waldor M. Barel M. Charbit A. Meibom K. L. (2009). Pivotal role of the *Francisella tularensis* heat-shock sigma factor RpoH. *Microbiology* 155 2560–2572. 10.1099/mic.0.029058-0 19443547 PMC2888120

[B24] Gross C. A. Chan C. Dombroski A. Gruber T. Sharp M. Tupy J. (1998). The functional and regulatory roles of sigma factors in transcription. *Cold Spring Harb. Symp. Quant. Biol.* 63 141–155. 10384278 10.1101/sqb.1998.63.141

[B25] Gruber T. M. Gross C. A. (2003). Multiple sigma subunits and the partitioning of bacterial transcription space. *Annu. Rev. Microbiol.* 57 441–466. 10.1146/annurev.micro.57.030502.090913 14527287

[B26] Guisbert E. Yura T. Rhodius V. A. Gross C. A. (2008). Convergence of molecular, modeling, and systems approaches for an understanding of the *Escherichia coli* heat shock response. *Microbiol. Mol. Biol. Rev.* 72 545–554. 10.1128/MMBR.00007-08 18772288 PMC2546862

[B27] Gunesekere I. C. Kahler C. M. Powell D. R. Snyder L. A. S. Saunders N. J. Rood J. I (2006). Comparison of the RpoH-dependent regulon and general stress response in *Neisseria gonorrhoeae*. *J. Bacteriol.* 188 4769–4776. 10.1128/jb.01807-05 16788186 PMC1483004

[B28] Han K. Li Z. F. Peng R. Zhu L. P. Zhou T. Wang L. G. (2013). Extraordinary expansion of a *Sorangium cellulosum* genome from an alkaline milieu. *Sci. Rep.* 3:2101. 10.1038/srep02101 23812535 PMC3696898

[B29] Hook-Barnard I. G. Hinton D. M. (2007). Transcription initiation by mix and match elements: flexibility for polymerase binding to bacterial promoters. *Gene Regul. Syst. Biol.* 1 275–293. 19119427 PMC2613000

[B30] Hook-Barnard I. G. Hinton D. M. (2009). The promoter spacer influences transcription initiation via σ70 region 1.1 of *Escherichia coli* RNA polymerase. *Proc. Natl. Acad. Sci. U.S.A.* 106 737–742. 10.1073/pnas.0808133106 19139410 PMC2630097

[B31] Ismail N. Bloch K. C. Mcbride J. W. (2010). Human ehrlichiosis and anaplasmosis. *Clin. Lab. Med.* 30 261–292. 10.1016/j.cll.2009.10.004 20513551 PMC2882064

[B32] Joo D. M. Ng N. Calendar R. (1997). A σ32 mutant with a single amino acid change in the highly conserved region 2.2 exhibits reduced core RNA polymerase affinity. *Proc. Natl. Acad. Sci. U.S.A.* 94 4907–4912. 10.1073/pnas.94.10.4907 9144163 PMC24604

[B33] Kill K. Binnewies T. T. Sicheritz-Pontén T. Willenbrock H. Hallin P. F. Wassenaar T. M. (2005). Genome update: sigma factors in 240 bacterial genomes. *Microbiology* 151 3147–3150. 10.1099/mic.0.28339-0 16207898

[B34] Kocan A. A. Levesque G. C. Whitworth L. C. Murphy G. L. Ewing S. A. Barker R. W. (2000). Naturally occurring *Ehrlichia chaffeensis* infection in coyotes from Oklahoma. *Emerg Infect. Dis.* 6 477–480. 10.3201/eid0605.000505 10998377 PMC2627953

[B35] Koo B. M. Rhodius V. A. Campbell E. A. Gross C. A. (2009a). Dissection of recognition determinants of *Escherichia coli* sigma32 suggests a composite -10 region with an ’extended -10’ motif and a core -10 element. *Mol. Microbiol.* 72 815–829. 10.1111/j.1365-2958.2009.06690.x 19400791 PMC4412615

[B36] Koo B. M. Rhodius V. A. Nonaka G. Dehaseth P. L. Gross C. A. (2009b). Reduced capacity of alternative σs to melt promoters ensures stringent promoter recognition. *Genes Dev.* 23 2426–2436. 10.1101/gad.1843709 19833768 PMC2764494

[B37] Kourennaia O. V. Tsujikawa L. Dehaseth P. L. (2005). Mutational analysis of *Escherichia coli* heat shock transcription factor sigma 32 reveals similarities with sigma 70 in recognition of the -35 promoter element and differences in promoter DNA melting and -10 recognition. *J. Bacteriol.* 187 6762–6769. 10.1128/jb.187.19.6762-6769.2005 16166539 PMC1251588

[B38] Kuriakose J. A. Miyashiro S. Luo T. Zhu B. Mcbride J. W. (2011). *Ehrlichia chaffeensis* transcriptome in mammalian and arthropod hosts reveals differential gene expression and post transcriptional regulation. *PLoS One* 6:e24136. 10.1371/journal.pone.0024136 21915290 PMC3167834

[B39] Larkin M. A. Blackshields G. Brown N. P. Chenna R. Mcgettigan P. A. Mcwilliam H. (2007). Clustal W and Clustal X version 2.0. *Bioinformatics* 23 2947–2948. 10.1093/bioinformatics/btm404 17846036

[B40] Liu H. Jakkula L. U. M. R. Von Ohlen T. Ganta R. R. (2016). Sequence determinants spanning -35 motif and AT-rich spacer region impacting *Ehrlichia chaffeensis* Sigma 70-dependent promoter activity of two differentially expressed p28 outer membrane protein genes. *DNA Res.* 23 495–505. 10.1093/dnares/dsw034 27402867 PMC5066175

[B41] Liu H. Von Ohlen T. Cheng C. Faburay B. Ganta R. R. (2013). Transcription of *Ehrlichia chaffeensis* genes is accomplished by RNA polymerase holoenzyme containing either sigma 32 or sigma 70. *PLoS One* 8:e81780. 10.1371/journal.pone.0081780 24278458 PMC3836757

[B42] Lockhart J. M. Davidson W. R. Stallknecht D. E. Dawson J. E. Little S. E. (1997). Natural history of *Ehrlichia chaffeensis* (Rickettsiales: Ehrlichieae) in the piedmont physiographic province of Georgia. *J. Parasitol.* 83 887–894. 9379294

[B43] Long S. W. Whitworth T. J. Walker D. H. Yu X. (2005). Overcoming barriers to the transformation of the genus *Ehrlichia*. *Ann. N. Y. Acad. Sci.* 1063 403–410. 10.1196/annals.1355.072 16481548

[B44] Martínez-Salazar J. M. Sandoval-Calderón M. Guo X. Castillo-Ramírez S. Reyes A. Loza M. G. (2009). The *Rhizobium etli* RpoH1 and RpoH2 sigma factors are involved in different stress responses. *Microbiology* 155 386–397. 10.1099/mic.0.021428-0 19202087

[B45] Mathews S. A. Douglas A. Sriprakash K. S. Hatch T. P. (1993). In vitro transcription in *Chlamydia psittaci* and *Chlamydia trachomatis*. *Mol. Microbiol.* 7 937–946. 10.1111/j.1365-2958.1993.tb01185.x 8483421

[B46] Matsui M. Takaya A. Yamamoto T. (2008). σ32 mediated negative regulation of *Salmonella* pathogenicity island 1 expression. *J. Bacteriol.* 190 6636–6645. 10.1128/JB.00744-08 18723621 PMC2566199

[B47] mcClure E. E. Chávez A. S. O. Shaw D. K. Carlyon J. A. Ganta R. R. Noh S. M. (2017). Engineering of obligate intracellular bacteria: progress, challenges and paradigms. *Nat. Rev. Microbiol.* 15 544–558. 10.1038/nrmicro.2017.59 28626230 PMC5557331

[B48] Meibom K. L. Dubail I. Dupuis M. Barel M. Lenco J. Stulik J. (2008). The heat-shock protein ClpB of *Francisella tularensis* is involved in stress tolerance and is required for multiplication in target organs of infected mice. *Mol. Microbiol.* 67 1384–1401. 10.1111/j.1365-2958.2008.06139.x 18284578

[B49] Mogk A. Tomoyasu T. Goloubinoff P. Rüdiger S. Röder D. Langen H. (1999). Identification of thermolabile *Escherichia coli* proteins: prevention and reversion of aggregation by DnaK and ClpB. *EMBO J.* 18 6934–6949. 10.1093/emboj/18.24.6934 10601016 PMC1171757

[B50] Mulligan M. E. Brosius J. Mcclure W. R. (1985). Characterization *in vitro* of the effect of spacer length on the activity of *Escherichia coli* RNA polymerase at the TAC promoter. *J. Biol. Chem.* 260 3529–3538. 3882710

[B51] Nishihara K. Kanemori M. Kitagawa M. Yanagi H. Yura T. (1998). Chaperone coexpression plasmids: differential and synergistic roles of DnaK-DnaJ-GrpE and GroEL-GroES in assisting folding of an allergen of Japanese cedar pollen, Cryj2, in *Escherichia coli*. *Appl. Environ. Microbiol.* 64 1694–1699. 9572938 10.1128/aem.64.5.1694-1699.1998 PMC106217

[B52] Nonaka G. Blankschien M. Herman C. Gross C. A. Rhodius V. A. (2006). Regulon and promoter analysis of the *E. coli* heat-shock factor, sigma32, reveals a multifaceted cellular response to heat stress. *Genes Dev.* 20 1776–1789. 10.1101/gad.1428206 16818608 PMC1522074

[B53] Paget M. S. Helmann J. D. (2003). The sigma70 family of sigma factors. *Genome Biol.* 4:203. 12540296 10.1186/gb-2003-4-1-203 PMC151288

[B54] Rao X. Deighan P. Hua Z. Hu X. Wang J. Luo M. (2009). A regulator from *Chlamydia trachomatis* modulates the activity of RNA polymerase through direct interaction with the beta subunit and the primary sigma subunit. *Genes Dev.* 23 1818–1829. 10.1101/gad.1784009 19651989 PMC2720258

[B55] Rikihisa Y. (2010). *Anaplasma phagocytophilum* and *Ehrlichia chaffeensis*: subversive manipulators of host cells. *Nat. Rev. Micro.* 8 328–339. 10.1038/nrmicro2318 20372158

[B56] Rud I. Jensen P. R. Naterstad K. Axelsson L. (2006). A synthetic promoter library for constitutive gene expression in Lactobacillus plantarum. *Microbiology* 152 1011–1019. 10.1099/mic.0.28599-0 16549665

[B57] Sahu G. K. Chowdhury R. Das J. (1994). Heat shock response and heat shock protein antigens of *Vibrio cholerae*. *Infect. Immun.* 62 5624–5631. 7960144 10.1128/iai.62.12.5624-5631.1994 PMC303311

[B58] Seo G. M. Cheng C. Tomich J. Ganta R. R. (2008). Total, membrane, and immunogenic proteomes of macrophage- and tick cell-derived *Ehrlichia chaffeensis* evaluated by liquid chromatography-tandem mass spectrometry and MALDI-TOF methods. *Infect. Immun.* 76 4823–4832. 10.1128/IAI.00484-08 18710870 PMC2573352

[B59] Shen L. Li M. Zhang Y. (2004). *Chlamydia trachomatis* σ28 recognizes the fliC promoter of *Escherichia coli* and responds to heat shock in chlamydiae. *Microbiology* 150 205–215. 10.1099/mic.0.26734-0 14702414

[B60] Shimada T. Yamazaki Y. Tanaka K. Ishihama A. (2014). The whole set of constitutive promoters recognized by RNA polymerase RpoD holoenzyme of *Escherichia coli*. *PLoS One* 9:e90447. 10.1371/journal.pone.0090447 24603758 PMC3946193

[B61] Singh S. S. Typas A. Hengge R. Grainger D. C. (2011). *Escherichia coli* sigma70 senses sequence and conformation of the promoter spacer region. *Nucleic Acids Res.* 39 5109–5118. 10.1093/nar/gkr080 21398630 PMC3130263

[B62] Slamti L. Livny J. Waldor M. K. (2007). Global gene expression and phenotypic analysis of a *Vibrio cholerae* rpoH deletion mutant. *J. Bacteriol.* 189 351–362. 10.1128/jb.01297-06 17085549 PMC1797412

[B63] Spector M. P. Kenyon W. J. (2012). Resistance and survival strategies of *Salmonella enterica* to environmental stresses. *Food Res. Int.* 45 455–481. 10.1016/j.foodres.2011.06.056

[B64] Stoll S. Feldhaar H. Gross R. (2009). Promoter characterization in the at-rich genome of the obligate endosymbiont “*Candidatus Blochmannia floridanus*”. *J. Bacteriol.* 191 3747–3751. 10.1128/JB.00069-09 19329646 PMC2681919

[B65] Su J. Yang J. Zhao D. Kawula T. H. Banas J. A. Zhang J.-R. (2007). Genome-wide identification of *Francisella tularensis* virulence determinants. *Infect. Immun.* 75 3089–3101. 10.1128/iai.01865-06 17420240 PMC1932872

[B66] Tan M. Engel J. N. (1996). Identification of sequences necessary for transcription *in vitro* from the *Chlamydia trachomatis* rRNA P1 promoter. *J. Bacteriol.* 178 6975–6982. 10.1128/jb.178.23.6975-6982.1996 8955322 PMC178601

[B67] Tan M. Gaal T. Gourse R. L. Engel J. N. (1998). Mutational analysis of the *Chlamydia trachomatis* rRNA P1 promoter defines four regions important for transcription in vitro. *J. Bacteriol.* 180 2359–2366. 9573186 10.1128/jb.180.9.2359-2366.1998 PMC107176

[B68] Tempel R. Lai X.-H. Crosa L. Kozlowicz B. Heffron F. (2006). Attenuated *Francisella novicida* transposon mutants protect mice against wild-type challenge. *Infect. Immun.* 74 5095–5105. 10.1128/iai.00598-06 16926401 PMC1594869

[B69] Tripathi L. Zhang Y. Lin Z. (2014). Bacterial sigma factors as targets for engineered or synthetic transcriptional control. *Front. Bioeng. Biotechnol.* 2:33. 10.3389/fbioe.2014.00033 25232540 PMC4153023

[B70] Unver A. Rikihisa Y. Stich R. W. Ohashi N. Felek S. (2002). The omp-1 major outer membrane multigene family of *Ehrlichia chaffeensis* is differentially expressed in canine and tick hosts. *Infect. Immun.* 70 4701–4704. 10.1128/iai.70.8.4701-4704.2002 12117987 PMC128143

[B71] Walker D. H. Dumler J. S. (1996). Emergence of the ehrlichioses as human health problems. *Emerg. Infect. Dis.* 2 18–29. 10.3201/eid0201.960102 8903194 PMC2639805

[B72] Walker D. H. Paddock C. D. Dumler J. S. (2008). Emerging and re-emerging tick-transmitted *rickettsial* and *ehrlichial* infections. *Med. Clin. North Am.* 92 1345–1361. 10.1016/j.mcna.2008.06.002 19061755

[B73] Wang Y. deHaseth P. L. (2003). Sigma 32-dependent promoter activity *in vivo*: sequence determinants of the *groE* promoter. *J. Bacteriol.* 185 5800–5806. 10.1128/jb.185.19.5800-5806.2003 13129951 PMC193967

[B74] Wang Y. Wei L. Liu H. Cheng C. Ganta R. R. (2017). A genetic system for targeted mutations to disrupt and restore genes in the obligate bacterium, *Ehrlichia chaffeensis*. *Sci. Rep.* 7:15801. 10.1038/s41598-017-16023-y 29150636 PMC5693922

[B75] Weiss D. S. Brotcke A. Henry T. Margolis J. J. Chan K. Monack D. M. (2007). In vivo negative selection screen identifies genes required for *Francisella virulence*. *Proc. Natl. Acad. Sci. U.S.A.* 104 6037–6042. 10.1073/pnas.0609675104 17389372 PMC1832217

[B76] Wood D. O. Wood R. R. Tucker A. M. (2014). Genetic systems for studying obligate intracellular pathogens: an update. *Curr. Opin. Microbiol.* 17 11–16. 10.1016/j.mib.2013.10.006 24581687 PMC3942670

[B77] Yabsley M. J. (2010). Natural history of *Ehrlichia chaffeensis*: vertebrate hosts and tick vectors from the United States and evidence for endemic transmission in other countries. *Vet. Parasitol.* 167 136–148. 10.1016/j.vetpar.2009.09.015 19819631

[B78] Yu H. H. Tan M. (2003). σ28 RNA polymerase regulates hctB, a late developmental gene in Chlamydia. *Mol. Microbiol.* 50 577–584. 10.1046/j.1365-2958.2003.03708.x 14617180 PMC2810255

[B79] Zhang J. Z. Popov V. L. Gao S. Walker D. H. Yu X. J. (2007). The developmental cycle of *Ehrlichia chaffeensis* in vertebrate cells. *Cell Microbiol.* 9 610–618. 10.1111/j.1462-5822.2006.00812.x 16987329

[B80] Zhang T. Kedzierska-Mieszkowska S. Liu H. Cheng C. Ganta R. R. Zolkiewski M. (2013). Aggregate-reactivation activity of the molecular chaperone ClpB from *Ehrlichia chaffeensis*. *PLoS One* 8:e62454. 10.1371/journal.pone.0062454 23667479 PMC3646808

[B81] Zhao K. Liu M. Burgess R. R. (2005). The global transcriptional response of *Escherichia coli* to induced sigma 32 protein involves sigma 32 regulon activation followed by inactivation and degradation of sigma 32 *in vivo*. *J. Biol. Chem.* 280 17758–17768. 15757896 10.1074/jbc.M500393200

