# Supplementary material for: Sequence Determinants Spanning −10 Motif and Spacer Region Implicated in Unique Ehrlichia chaffeensis Sigma 32-Dependent Promoter Activity of dnaK Gene
Source: Front Microbiol. 2019 Aug 2;10:1772. doi: 10.3389/fmicb.2019.01772 (PMC6687850; doi:10.3389/fmicb.2019.01772)
Supplement: Supplementary file 1 [file Data_Sheet_1.PDF]

	-35		-10	+1	
dnaK **(WT)** ⟶	CATAAATTTATGAACTTGTGTTAAATGGGG<u>TTGTAA</u>	**TCTTATGATTTGGTTAT**	<u>TATATC</u>TGTGATT	<u>A</u>	TAGTTAA

	-35		-10	+1	
dnaK **(0bp)** ⟶	CATAAATTTATGAAC<u>*TTGTGT*</u>	***TAAATGGGGTTGTAA***^^^^^^^^^^^^^^^^^	<u>TATATC</u>TGTGATT	<u>A</u>	TAGTTAA

Supplementary Figure S1 *dnaK* sequence used for wild type promoter (WT) and for the complete deletion of spacer sequence (0 bp). The transcription start site (+1), -10 and -35 motifs were identified with underlined text. Spacer sequence was identified as bold text. Alternate predicted -35 and spacer sequences for complete spacer sequence deletion were identified in the 0 bp sequence. The symbol ^ refers to the deletion segment from WT *dnaK*.
