# Supplementary material for: Sequence Determinants Spanning −10 Motif and Spacer Region Implicated in Unique Ehrlichia chaffeensis Sigma 32-Dependent Promoter Activity of dnaK Gene
Source: Front Microbiol. 2019 Aug 2;10:1772. doi: 10.3389/fmicb.2019.01772 (PMC6687850; doi:10.3389/fmicb.2019.01772)
Supplement: Supplementary file 2 [file Data_Sheet_2.PDF]

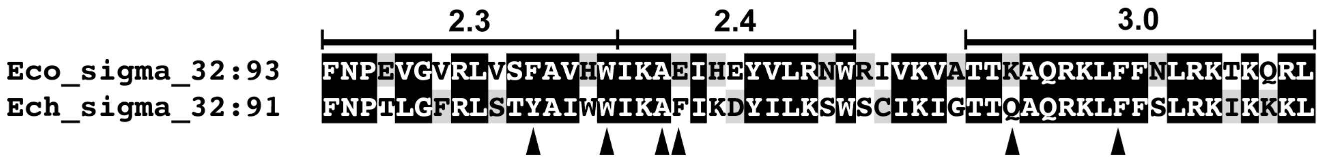

Supplementary Figure S2 Amino acid sequence alignment of *E. coli* σ^{32} (Eco_sigma_32), and *E. chaffeensis* σ^{32} (Eco_sigma_32) for the regions 2.3 to 3.0. Regions that are conserved among eubacteria sigma factors are denoted by the number above the sequences. Black triangles refer to the amino acids where substitution mutations were performed.
