# Supplementary material for: Sequence Determinants Spanning −10 Motif and Spacer Region Implicated in Unique Ehrlichia chaffeensis Sigma 32-Dependent Promoter Activity of dnaK Gene
Source: Front Microbiol. 2019 Aug 2;10:1772. doi: 10.3389/fmicb.2019.01772 (PMC6687850; doi:10.3389/fmicb.2019.01772)
Supplement: Supplementary file 3 [file Table_1.DOC]

**Supplementary Table S1** Bacterial strains and plasmids used in this study

| Name | Description | References |
| --- | --- | --- |
| *E. coli* |  |  |
| Top10 | Δ*lac*Χ74 *rec*A1 *ara*D139 | Invitrogen |
|  | 7697 *gal*U *gal*K *rps*L (StrR) *end*A1 *nup*G |  |
| CAG57101 | MG1655 △lacX74 PBAD_groESL::cat △rpoH::aadA, CmR, SpR, | ([Koo et al., 2009a](#_ENREF_36)) |
|  |  |  |
| Plasmids | | |
| pSAKT-Eco_rpoH | p15A ori, P_lac_, *E. coli rpoH*, *lacI*^q^, Amp^R^ (pSAKT32)**^#^** | ([Koo et al., 2009a](#_ENREF_36)) |
| pSAKT-Ech_rpoH | For *E. chaffeensis* σ^32^ expression (pSAKT32-Ech_rpoH)**^#^** | ([Liu et al., 2013](#_ENREF_42)) |
| pSAKT | pSAKT blank vector | This study |
| pSAKT-Ech_rpoH_Y102A | σ^32^- Y102A in pSAKT-Ech_rpoH | This study |
| pSAKT-Ech_rpoH_W106A | σ^32^- W106A in pSAKT-Ech_rpoH | This study |
| pSAKT-Ech_rpoH_A109Q pSAKT32-Ech_rpoH This study | σ^32^- A109Q in pSAKT-Ech_rpoH | This study |
| pSAKT-Ech_rpoH_F110A | σ^32^- F110A in pSAKT-Ech_rpoH | This study |
| pSAKT-Ech_rpoH_F110E | σ^32^- F110E in pSAKT-Ech_rpoH | This study |
| pSAKT-Ech_rpoH_Q128A pSAKT32-Ech_rpoH This study | σ^32^- Q128A in pSAKT-Ech_rpoH | This study |
| pSAKT-Ech_rpoH_F134A | σ^32^- F134A in pSAKT-Ech_rpoH | This study |
| pET32-Ech_rpoH | For overexpression of *E. chaffeensis* σ^32^ | ([Liu et al., 2013](#_ENREF_42)) |
| pQF50K | pQF50K plasmid without promoter | ([Liu et al., 2013](#_ENREF_42)) |
| pQF50K-Ech_dnaK | *E. chaffeensis dnaK* promoter in pQF50K | ([Liu et al., 2013](#_ENREF_42)) |
| pQF50K-Ech_dnaK-35 | Deletion of -35 motif | ([Liu et al., 2013](#_ENREF_42)) |
| pQF50K-Ech_dnaK-10 | Deletion of -10 motif | This study |
| pQF50K-Ech_dnaK_T1G | *dnaK* promoter (T1G) in pQF50K-dnaK | This study |
| pQF50K-Ech_dnaK_T1C | *dnaK* promoter (T1C) in pQF50K-dnaK | This study |
| pQF50K-Ech_dnaK_T1A | *dnaK* promoter (T1A) in pQF50K-dnaK | This study |
| pQF50K-Ech_dnaK_A2C | dnaK promoter (A2C) in pQF50K-dnaK | This study |
| pQF50K-Ech_dnaK_A2T | *dnaK* promoter (A2T) in pQF50K-dnaK | This study |
| pQF50K-Ech_dnaK_A2G | *dnaK* promoter (A2G) in pQF50K-dnaK | This study |
| pQF50K-Ech_dnaK_T3G | *dnaK* promoter (T3G) in pQF50K-dnaK | This study |
| pQF50K-Ech_dnaK_T3C | *dnaK* promoter (T3C) in pQF50K-dnaK | This study |
| pQF50K-Ech_dnaK_T3A | *dnaK* promoter (T3A) in pQF50K-dnaK | This study |
| pQF50K-Ech_dnaK_A4G  pQF50K-dnaK_T3C  pQF50K-dnaK_T3A | *dnaK* promoter (A4G) in pQF50K-dnaK | This study |
| pQF50K-Ech_dnaK_A4C  pQF50K-dnaK_T3C  pQF50K-dnaK_T3A | *dnaK* promoter (A4C) in pQF50K-dnaK | This study |
| pQF50K-Ech_dnaK_A4T  pQF50K-dnaK_T3C  pQF50K-dnaK_T3A | *dnaK* promoter (A4T) in pQF50K-dnaK | This study |
| pQF50K-Ech_dnaK_T5C | *dnaK* promoter (T5C) in pQF50K-dnaK | This study |
| pQF50K-Ech_dnaK_T5A | *dnaK* promoter (T5A) in pQF50K-dnaK | This study |
| pQF50K-Ech_dnaK_T5G | *dnaK* promoter (T5G) in pQF50K-dnaK | This study |
| pQF50K-Ech_dnaK_C6G | *dnaK* promoter (C6G) in pQF50K-dnaK | This study |
| pQF50K-Ech_dnaK_C6T | *dnaK* promoter (C6T) in pQF50K-dnaK | This study |
| pQF50K-Ech_dnaK_C6A | *dnaK* promoter (C6A) in pQF50K-dnaK | This study |

| Name | Description | Reference |
| --- | --- | --- |
| pQF50K-Ech_dnaK_2C | **TATTATATC mutated to TACCATATC** | This study |
| pQF50K-Ech_dnaK_4C | **TATTATATC mutated to CCCCATATC** | This study |
| pQF50K-Ech_dnaK_CP | *dnaK* promoter with complementary spacer | This study |
| pQF50K-Ech_dnaK_GC | *dnaK* promoter with GC-rich spacer | This study |
| pQF50K-Ech_dnaK_14bp | *dnaK* promoter with 14 bp spacer | This study |
| pQF50K-Ech_dnaK_15bp | *dnaK* promoter with 15 bp spacer | This study |
| pQF50K-Ech_dnaK_16bp | *dnaK* promoter with 16 bp spacer | This study |
| pQF50K-Ech_dnaK_18bp | *dnaK* promoter with 18 bp spacer | This study |
| pQF50K-Ech_dnaK_19bp | *dnaK* promoter with 19 bp spacer | This study |
| pQF50K-Ech_dnaK_20bp | *dnaK* promoter with 20 bp spacer | This study |
| pQF50K-Ech_dnaK_0bp | Deletion of entire 17 bp spacer | This study |
| pMT504 | Amp^R^; as templates of *in vitro* transcription | ([Tan and Engel, 1996](#_ENREF_67)) |
| pMT504-Ech_dnaK | *E. chaffeensis* *dnak* promoter in pMT504 | ([Liu et al., 2013](#_ENREF_42)) |
| pMT504-Ech_dnaK_T1G | *dnaK* promoter (T1G) in pMT504-dnaK | This study |
| pMT504-Ech_dnaK_T1A | *dnaK* promoter (T1A) in pMT504-dnaK | This study |
| pMT504-Ech_dnaK_A2G | *dnaK* promoter (A2G) in pMT504-dnaK | This study |
| pMT504-Ech_dnaK_T5A | *dnaK* promoter (T5A) in pMT504-dnaK | This study |
| pMT504-Ech_dnaK_C6T | *dnaK* promoter (C6T) in pMT504-dnaK | This study |
|  | | |

**Supplementary Table S1** Bacterial strains and plasmids used in this study (continued)

# The names in bracket is previously shown at references.
