# Supplementary material for: Sequence Determinants Spanning −10 Motif and Spacer Region Implicated in Unique Ehrlichia chaffeensis Sigma 32-Dependent Promoter Activity of dnaK Gene
Source: Front Microbiol. 2019 Aug 2;10:1772. doi: 10.3389/fmicb.2019.01772 (PMC6687850; doi:10.3389/fmicb.2019.01772)
Supplement: Supplementary file 4 [file Table_2.DOC]

| Number | Sequences | Orientation | Use |
| --- | --- | --- | --- |
|  | |  |  |
| For -10 motif (TATATC) mutagenesis of *E. chaffeensis dnaK* promoter in pQF50K-Ech_dnaK | |  |  |
|  |  |  |  |
| RRG1806 | 5'-atttggttat**AATATC**tgtgattatagttaatc-3' | Forward | T1 change to A |
| RRG1808 | 5'-atttggttat**CATATC**tgtgattatagttaatc-3' | Forward | T1 change to C |
| RRG1809 | 5'-atttggttat**GATATC**tgtgattatagttaatc-3' | Forward | T1 change to G |
| RRG1807 | 5'-cataagattacaaccccatttaac-3' | Reverse | T1 change to A or C or G |
| RRG1810 | 5'-tttggttat**TTTATC**tgtgattatagttaatc-3' | Forward | A2 change to T |
| RRG1812 | 5'-tttggttat**TCTATC**tgtgattatagttaatc-3' | Forward | A2 change to C |
| RRG1813 | 5'-tttggttat**TGTATC**tgtgattatagttaatc-3' | Forward | A2 change to G |
| RRG1811 | 5'-tcataagattacaaccccatttaac-3' | Reverse | A2 change to T or C or G |
| RRG1814 | 5'-ttggttat**TAAATC**tgtgattatagttaatc-3' | Forward | T3 change to A |
| RRG1816 | 5'-ttggttat**TACATC**tgtgattatagttaatc-3' | Forward | T3 change to C |
| RRG1817 | 5'-ttggttat**TAGATC**tgtgattatagttaatc-3' | Forward | T3 change to G |
| RRG1815 | 5'-atcataagattacaaccccatttaac-3' | Reverse | T3 change to A or C or G |
| RRG1853 | 5'-tggttat**TATTTC**tgtgattatagttaatc-3' | Forward | A4 change to T |
| RRG1854 | 5'-tggttat**TATCTC**tgtgattatagttaatc-3' | Forward | A4 change to C |
| RRG1855 | 5'-tggttat**TATGTC**tgtgattatagttaatc -3' | Forward | A4 change to G |
| RRG1856 | 5'-aatcataagattacaacccc-3' | Reverse | A4 change to T or C or G |
|  |  |  |  |

**Supplementary Table S2** Oligonucleotides use in this study

**Supplementary Table S2** Oligonucleotides use in this study (continued)

| Number | Sequences | Orientation | | | Use | |  |  | |  |
| --- | --- | --- | --- | --- | --- | --- | --- | --- | --- | --- |
|  |  |  | |  | |  | | |  | |
| For -10 motif (TATATC) mutagenesis of *E. chaffeensis dnaK* promoter in pQF50K-Ech_dnaK | |  | |  | |  | | |  | |
|  |  |  | |  | |  | | |  | |
| RRG1857 | 5'-ggttat**TATAAC**tgtgattatagttaatctag -3' | Forward | | T5 change to A | | | | |  | |
| RRG1858 | 5'-ggttat**TATACC**tgtgattatagttaatc-3' | Forward | | T5 change to C | | | | |  | |
| RRG1859 | 5'-ggttat**TATAGC**tgtgattatagttaatc-3' | Forward | | T5 change to G | | | | |  | |
| RRG1860 | 5'-aaatcataagattacaaccc-3' | Reverse | | T5 change to T or C or G | | | | |  | |
| RRG1861 | 5'-gttat**TATATA**tgtgattatagttaatctagaag-3' | Forward | | C6 change to A | | | | |  | |
| RRG1862 | 5'-gttat**TATATT**tgtgattatagttaatctagaag-3' | Forward | | C6 change to T | | | | |  | |
| RRG1863 | 5'-gttat**TATATG**tgtgattatagttaatctag-3' | Forward | | C6 change to G | | | | |  | |
| RRG1864 | 5'-caaatcataagattacaacccc-3' | Reverse | | C6 change to T or A or G | | | | |  | |
|  |  |  | |  | | | | |  | |
| For substitutions in regions 2.3, 2.4 and 3.0 of *E. chaffeensis* σ^32^ in pSAKT-Ech_rpoH | |  | |  | | | | |  | |
|  |  |  | |  | | | | |  | |
| RRG1839 | 5'-gttatccact**GCG**gctatttggtggatc-3' | Forward | | Substitution Y102A | | | | |  |  |
| RRG1840 | 5'-ctaaagccaagagtgggattg-3' | Reverse | | Substitution Y102A | | | | |  |  |
| RRG1834 | 5'-tgctatttgg**GC**gatcaaagcttttattaaggac-3' | Forward | | Substitution W106A | | | | |  | |
| RRG1835 | 5'-taagtggataacctaaagc-3' | Reverse | | Substitution W106A | | | | |  | |
| RRG1841 | 5’-gtggatcaaa**CAG**tttattaaggactatattcttaaatcttg-3’ | Forward |  | Substitution A109Q | | | |  |  |  |
| RRG1842 | 5’-caaatagcataagtggataac-3’ | Reverse |  | Substitution A109Q | | | |  |  |  |

**Supplementary Table S2** Oligonucleotides use in this study (continued)

| Number | Sequences | | Orientation | | | | | | Use |  | | | | | | |  |
| --- | --- | --- | --- | --- | --- | --- | --- | --- | --- | --- | --- | --- | --- | --- | --- | --- | --- |
|  |  | |  | | | | |  |  | | | |  | | |  |  |
| RRG1836 | 5'-gatcaaagct**GCG**attaaggactatattcttaaatc-3' | Forward | | | Substitution F110A | | | | | |  |  |  |  |  |  |  |
| RRG1837 | 5'-gatcaaagct**GAA**attaaggactatattcttaaatc-3' | Forward | | | Substitution F110E | | | | | | | | |  |  |  |  |
| RRG1838 | 5'-caccaaatagcataagtgg-3' | Reverse | | | Substitution F110A or F110E | | | | | | | | |  |  |  |  |
| RRG1843 | 5’-tggtacaaca**GCG**gcacaaaggaagttattc-3’ | Forward | |  | | Substitution Q128A | | | | |  | | | |  |  |  |
| RRG1844 | 5’-attttaatgcacgaccaag-3’ | Reverse | |  | | Substitution Q128A | | | | |  | | | |  |  |  |
| RRG1845 | 5’-tta**GCG**tttagcttaaggaaaattaagaaaaaactttttaaatataac-3’ | Forward | |  | | Substitution F134A | | | | |  | | | |  |  |  |
| RRG1846 | 5’-cttcctttgtgcttgtgttgtac-3’ | Reverse | |  | | Substitution F134A | | | | |  | | | |  |  |  |
| For mutations in spacer of *E. chaffeensis* *dnaK* promoter in pQF50K-Ech_dnaK | |  | | | | |  | | | | | |  | | |  |  |
|  |  |  | | | | |  | | | | | |  | | |  |  |
| RRG 1699 | 5’-aaccaatatatatctgtgattatagttaatctag-3’ | Forward | | | | | Complementary spacer | | | | | |  | | |  |  |
| RRG 1700 | 5’-tagtattctttacaaccccatttaacac-3’ | Reverse | | | | |  | | | | | |  | | |  |  |
| RRG1705 | 5’-ccggttattatatctgtgattatagttaatctag-3’ | Forward | | | | | GC-rich Spacer | | | | | |  | | |  |  |
| RRG1706 | 5’-accctcggattacaaccccatttaacac-3’ | Reverse | | | | |  | | | | | |  | | |  |  |
| RRG1707  RRG1709 | 5’-atggttattatatctgtgattatagttaatc-3’  5’-aatcataagattacaaccccatttaac-3’ | Forward  Reverse | | | | | The length of spacer  :18 bp | | | | | |  | | |  |  |
| RRG1710  RRG1709 | 5’-aatggttattatatctgtgattatagttaatc-3’ | Forward  Reverse | | | | | The length of spacer  :19 bp | | | | |  |  |  |  |  |  |
| RRG1711  RRG1709 | 5’-atatggttattatatctgtgattatagttaatc-3’ | Forward  Reverse | | | | | The length of spacer  :20 bp | | | | |  |  |  |  |  |  |
| RRG1712  RRG1713  RRG1714  RRG1713 | 5’-ttggttattatatctgtgattatag-3’  5’-tcataagattacaaccccatttaac-3’  5’-tggttattatatctgtgattatagttaatc-3’ | Forward  Reverse  Forward  Reverse | | | | | The length of spacer  :16 bp  The length of spacer  :15 bp | | | | |  |  |  |  |  |  |

**Supplementary Table S2** Oligonucleotides use in this study (continued)

| Number | Sequences | Orientation | | Use | |  |  |
| --- | --- | --- | --- | --- | --- | --- | --- |
|  |  |  |  | |  | |  |
| For mutations in spacer of *E. chaffeensis* *dnaK* promoter in pQF50K-Ech_dnaK | |  |  | |  | |  |
|  |  |  |  | |  | |  |
| RRG1714  RRG1715 | 5’-cataagattacaaccccatttaac-3’ | Forward  Reverse | The length of spacer  :14 bp | | | |  |
| RRG1716  RRG1717 | 5’-tatatctgtgattatagttaatctag-3’  5’-ttacaaccccatttaacac-3’ | Forward  Reverse | Deletion of all spacer | | | |  |
|  |  |  |  | | | |  |
| For -10 motif (TATATC) mutagenesis of *E. chaffeensis* *dnaK* promoter in pMT504-Ech_dnaK | |  |  | | | |  |
|  |  |  |  | | | |  |
| RG302  RRG1807 | 5’-atttggttat**GATATC**tgtgattataatc-3’ | Forward  Reverse | T1 change to G | | | |  |
| RG303  RRG1807 | 5’-atttggttat**AATATC**tgtgattataatc-3’ | Forward  Reverse | T1 change to A | | | |  |
| RG304  RG305 | 5’-tttggttat**TGTATC**tgtgattataatc-3’  5’-tcataagattacaacccc-3’ | Forward  Reverse | A2 change to G | | | |  |
| RG307  RRG1860 | 5’-ggttat**TATAAC**tgtgattataatctttaaac-3’ | Forward  Reverse | T5 change to A | | | |  |
| RG308  RRG1864 | 5’-gttat**TATATT**tgtgattataatctttaaacac-3’ | Forward  Reverse | C6 change to T | | | |  |

**Supplementary Table S2** Oligonucleotides use in this study (continued)

| Number | Sequences | Orientation | | Use | |  |  | |
| --- | --- | --- | --- | --- | --- | --- | --- | --- |
|  |  |  |  | |  | | |  |
| For deletion for -10 motif of *E. chaffeensis* *dnaK* promoter in pQF50K-Ech_dnaK | |  |  | | | | |  |
|  |  |  |  | | | | |  |
| DnaK-10F  DnaK-10R | 5’-tgtgattatagttaatctagaag-3’  5’-ataaccaaatcataagattacaac-3’ | Forward  Reverse |  | | | | |  |
|  |  |  |  | | | | |  |
| For preparation of pSAKT blank vector | |  |  | | | | |  |
|  |  |  |  | | | | |  |
| RRG1656 | 5’-tcggtaataaccagaagaatgggg-3’ | Forward |  | | | | |  |
| RRG1655 | 5’-ggatccgcgacccatttg-3’ | Reverse |  | | | | |  |
|  |  |  |  | | | | |  |
| For 2C and 4C mutations of *E. chaffeensis dnaK* promoter in pQF50K-Ech_dnaK | |  |  | | | | |  |
|  |  |  |  | | | | |  |
| RRG2113  RRG2114 | 5’-gatttggtta**CCATATC**tgtgattatagttaatctag-3’  5’-ataagattacaaccccatttaac-3’ | Forward  Reverse | TT change to CC | | | | |  |
| RRG2115  RRG2116 | 5’-atgatttggt**CCCCATATC**tgtgattatagttaatc-3’  5’-aagattacaaccccatttaac-3’ | Forward  Reverse | TATT change to CCCC | | | | |  |
