# Supplementary material for: Sequence Determinants Spanning −10 Motif and Spacer Region Implicated in Unique Ehrlichia chaffeensis Sigma 32-Dependent Promoter Activity of dnaK Gene
Source: Front Microbiol. 2019 Aug 2;10:1772. doi: 10.3389/fmicb.2019.01772 (PMC6687850; doi:10.3389/fmicb.2019.01772)
Supplement: Supplementary file 5 [file Table_3.DOC]

| Bacteria | Consensus Sequence (-35 and -10 motif) | *dnak* promoter (-35, Spacer, -10 motif) | Class | References |
| --- | --- | --- | --- | --- |
| *Rhodobacter sphaeroides* |  | CGCTTGCAGccccaaatcccccccccTATATACGCCCG | α-proteobacteria | ([1](#_ENREF_1)) |
| *Acetobacter pasteurianus NBRC3283* | CTTG-(17/18 bp) ----- CYTAT-T--G | CTTGaaaaagagaaaacgccacCATACATCAG | α-proteobacteria | ([2](#_ENREF_2)) |
| *Caulobacter crescentus* |  | gcc**TT**gcgtggcggcccctatcccCaTATCCggctt cg | α-proteobacteria | ([3](#_ENREF_3)) |
| *Ehrlichia chaffeensis* | TTGAAA----TATATN | TTGTAAtcttatgatttggttatTATATCTGTGATTA | α-proteobacteria | ([4](#_ENREF_4)) |
| Candidatus Blochmannia floridanus | STTGAAT-----CCCCATAT | GTTGAAAagtacagttttggCCGCATAT | γ-proteobacteria | ([5](#_ENREF_5)) |
| Vibrio cholerae | cTTGAA(N(_13-16_)(a/c)CCATat(a/t) | GTTGAAAacccgattctccatCCCCACATTAGGGGTA | γ-proteobacteria | ([6](#_ENREF_6)) |
| *Franscisella tularensis LVS* | CTTGAAA---N(_13-16_)---CCATATA | CTTGAAAagattataaatatgcCCATCTA | γ-proteobacteria | ([7](#_ENREF_7)) |
| *Escherichia coli* | TTGAAA---N14-CCCCATWT | CTTGATGacgtggtttacgaCCCCATTTAGTAGTCA | γ-proteobacteria | ([8](#_ENREF_8)) |
| Neisseria gonorrhoeae | CTTGaaatt---(N_12-20_)---ccNNatttt | GCTGTAGCTTGAAAcagcccgccgcccg**CC**CT**ATTT**A | β-proteobacteria | ([9](#_ENREF_9)) |
| *Geobacter sulfurreducens* | CCCCCTTGA-------TGGTTACNTTA | CGG**CCCCTTGC**ttttggggagggcgaTGACTACGTTGTTTCC | [δ-proteobacteria](https://en.wikipedia.org/wiki/Deltaproteobacteria) | ([10](#_ENREF_10)) |

**Supplementary Table S3** Sigma 32-dependent promoters in proteobacteria

**1. Nuss, A.M., Glaeser, J. and Klug, G. (2009) RpoHII Activates Oxidative-Stress Defense Systems and Is Controlled by RpoE in the Singlet Oxygen-Dependent Response in Rhodobacter sphaeroides. *Journal of Bacteriology*, 191, 220-230.**

**2. Okamoto-Kainuma, A., Ishikawa, M., Nakamura, H., Fukazawa, S., Tanaka, N., Yamagami, K. and Koizumi, Y. (2011) Characterization of rpoH in Acetobacter pasteurianus NBRC3283. *J Biosci Bioeng*, 111, 429-432.**

**3. Reisenauer, A., Mohr, C.D. and Shapiro, L. (1996) Regulation of a heat shock sigma32 homolog in Caulobacter crescentus. *Journal of Bacteriology*, 178, 1919-1927.**

**4. Liu, H., Von Ohlen, T., Cheng, C., Faburay, B. and Ganta, R.R. (2013) Transcription of *Ehrlichia chaffeensis* Genes Is Accomplished by RNA Polymerase Holoenzyme Containing either Sigma 32 or Sigma 70. *PLoS ONE*, 8, e81780.**

**5. Stoll, S., Feldhaar, H. and Gross, R. (2009) Promoter Characterization in the AT-Rich Genome of the Obligate Endosymbiont “Candidatus Blochmannia floridanus”. *Journal of Bacteriology*, 191, 3747-3751.**

**6. Slamti, L., Livny, J. and Waldor, M.K. (2007) Global Gene Expression and Phenotypic Analysis of a Vibrio cholerae rpoH Deletion Mutant. *Journal of Bacteriology*, 189, 351-362.**

**7. Grall, N., Livny, J., Waldor, M., Barel, M., Charbit, A. and Meibom, K.L. (2009) Pivotal role of the *Francisella tularensis* heat-shock sigma factor RpoH. *Microbiology*, 155, 2560-2572.**

**8. Nonaka, G., Blankschien, M., Herman, C., Gross, C.A. and Rhodius, V.A. (2006) Regulon and promoter analysis of the *E. coli* heat-shock factor, sigma32, reveals a multifaceted cellular response to heat stress. *Genes Dev*, 20, 1776-1789.**

**9. Gunesekere, I.C., Kahler, C.M., Powell, D.R., Snyder, L.A.S., Saunders, N.J., Rood, J.I. and Davies, J.K. (2006) Comparison of the RpoH-Dependent Regulon and General Stress Response in Neisseria gonorrhoeae. *Journal of Bacteriology*, 188, 4769-4776.**

**10. Ueki, T. and Lovley, D.R. (2007) Heat-shock sigma factor RpoH from *Geobacter sulfurreducens*. *Microbiology*, 153, 838-846.**
